# Experimental and Theoretical Study on the Fatigue Crack Propagation in Stud Shear Connectors

**DOI:** 10.3390/ma16020701

**Published:** 2023-01-11

**Authors:** Yachuan Kuang, Yameng Wang, Ping Xiang, Li Tao, Kun Wang, Fan Fan, Jiahui Yang

**Affiliations:** School of Civil Engineering, Central South University, Changsha 410075, China

**Keywords:** stud shear connectors, fatigue test, fatigue properties, residual bearing capacity, fatigue crack propagation, numerically simulation

## Abstract

Steel-concrete composite girder bridges are subjected to reciprocal cyclic loading from vehicles, and the stud shear connectors are the key components for transmitting shear forces. Thus, it is necessary to study the fatigue performance of the stud shear connectors. At present, there are few studies on the fatigue crack propagation process of studs, and the variation curve of the crack depth of studs with the number of fatigue loading cycles is not clear. In this study, the degradation law of fatigue properties and the fatigue crack propagation law of stud shear connectors in steel-concrete composite structures are examined under fatigue loading. The fatigue properties, i.e., failure mode, the dynamic slip-fatigue number curve, cross-sectional characteristics, and the residual bearing capacity of the stud specimens, are first systematically studied through ten standard push-out specimen tests. The test results show that the relative value of the fatigue crack extension area increases, while the relative value of the residual bearing capacity of the studs decreases approximately linearly. Then, the expression of the relationship between the fatigue crack depth and the residual load-bearing capacity of the stud is proposed, based on the fatigue crack theory of fracture mechanics. Finally, combined with the ABAQUS and FRANC3D software, a fatigue crack propagation finite element analysis (FEA) model of the stud is established. The FEA results showed that the trends in the number of cyclic loads and the fatigue crack depth of studs are basically the same for the simulation curve, test curve and theoretical calculation curve.

## 1. Introduction

Steel-concrete composite structures are high-performance structures that combine the benefits of both concrete and steel. Owing to their high load-carrying capacity, high structural stiffness, and excellent ductility, such structures are widely used in high-rise buildings, heavy industrial buildings, and bridge construction. In practical engineering, steel-concrete composite structures, especially the composite girder bridges in railroad systems, are subjected to reciprocal cyclic loading from vehicles, so their fatigue performance analysis is of great practical significance.

The fatigue performance of studs, which are frequently applied as shear connectors and are the key components for transmitting shear forces in steel-concrete composite structures, has a significant influence on the fatigue life of composite structures. The fatigue test results of a large number of push-out specimens have suggested that the fatigue life of studs, measured by the push-out test, is lower than that when measured by the beam test. Therefore, the results of the push-out test are typically used internationally as the basis for the fatigue design of stud shear connectors [1,2]. Xu et al. [3] found that root fracture is the main fatigue failure mode of shear studs under fatigue loading. Huang et al. [4] demonstrated that all the residual mechanical indexes of the stud in push-out specimens are non-linearly degraded under fatigue loading. Hanswille et al. [5] proposed an expression for the residual bearing capacity and fatigue extended area of studs, but it was difficult to measure the fatigue extended area of studs accurately in practical engineering using this expression. Liu et al. [6] conducted accelerated corrosion push-out tests and proposed a fatigue life prediction model for studs, reflecting the effects of corrosion. Wei et al. [7] established the S-N curves and the prediction formulas for the evolution of plastic slip and elastic stiffness, via fatigue tests.

At present, only a few experimental studies have focused on the fatigue crack propagation law of studs, and the fatigue life and critical crack size of studs are generally analyzed by introducing an initial crack size and crack propagation rate equation, based on finite element simulation. Through finite element analysis (FEA), Wang et al. [8] showed that the initial crack size and the fatigue crack propagation rate equation have a significant impact on the fatigue life of stud shear connectors. Liang et al. [9] established a stud load slip model, considering the accumulation of slip, and it was observed that the slip accumulation of studs can be divided into three stages: “fast–slow–fast” stages. Several researchers have analyzed the fatigue crack propagation process, based on the fracture mechanics theory, and have obtained various fatigue crack propagation rate models for different materials and expansion stages [10,11,12].

At present, the fatigue crack propagation rate equation, adopted by scholars in finite element simulation, has not taken into account the influence of stress ratio upon specimens during loading; in addition, there are few studies on the fatigue crack propagation process of stud from a microscopic perspective. In addition, there are few studies on the degradation law of the residual bearing capacity of studs after a certain number of pre-fatigue loading cycles. In this paper, the push-out specimen, with a certain number of pre-fatigue loading cycles, was completely unloaded and changed to static loading until the specimen was damaged. Based on this method, the degradation law of the residual bearing capacity of the stud was studied. At the same time, the fatigue crack propagation rate equation, considering the stress ratio, was introduced. In addition, FRANC3D software was used to calculate the stress intensity factor on the leading edge of the crack nodes by the M-integral method, in order to analyze the change law of the crack tip stress field during the crack propagation process; the variation law of the fatigue crack propagation rate of studs was revealed from a microscopic perspective.

In this study, the fatigue performance, fatigue section characteristics, and the crack size of stud shear connectors are examined via experimental tests and FEA. An expression of the relationship between the fatigue crack depth and the residual bearing capacity of the stud is established, based on the fatigue crack theory of fracture mechanics. ABAQUS software is used to establish the FEA model of the push-out specimen, and combined with FRANC3D software, it is used to analyze the fatigue crack propagation process of the stud. Furthermore, the numerically simulated (based on FEA), theoretical, and experimental values for the crack depth of the stud, under different numbers of cyclic loads, are compared. Table 1 summarizes the abbreviations that appear throughout the article.

## 2. Specimen Design and Loading Test of Stud Shear Connectors

### 2.1. Specimen Design and Fabrication

**Figure 1:**
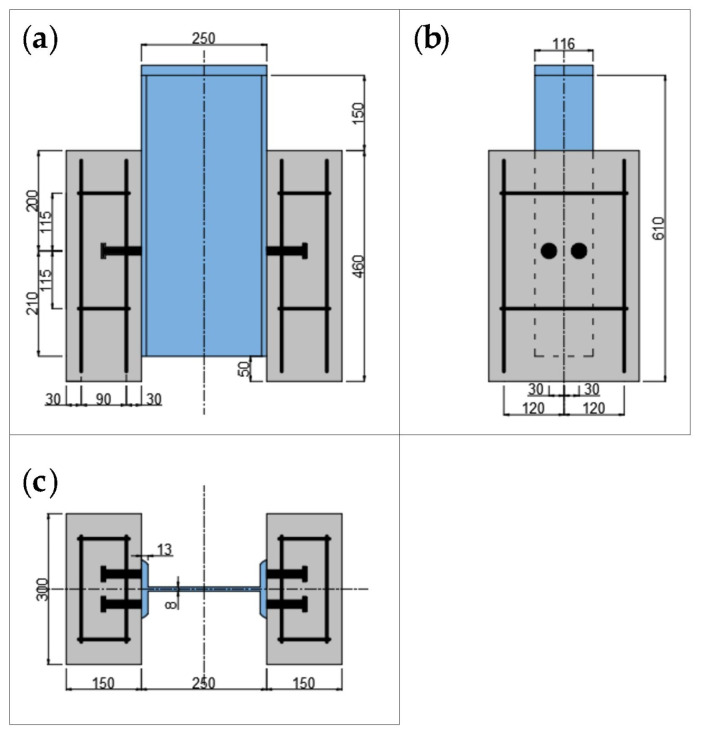
Detailed size of push-out specimen (unit: mm): (**a**) main view; (**b**) left view; (**c**) top view.

**Figure 2:**
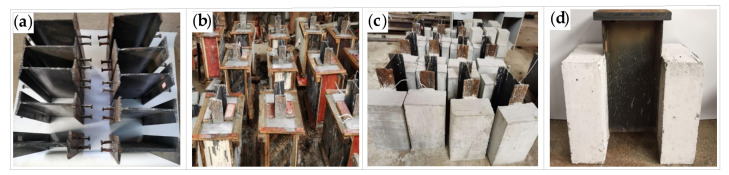
Fabrication of push-out specimen: (**a**) welding studs; (**b**) concrete pouring; (**c**) concrete curing; (**d**) welding steel plates.

The specimen design was based on the push-out specimen size and reinforcement recommended by Eurocode 4 [13], and the detailed dimensions of the specimen are shown in Figure 1. A total of 10 push-out specimens were made, including two specimens for static tests and eight specimens for the fatigue test. The steel beams were I-beam with Q345 grade; the size of the I-beam was 250 × 116 × 8 mm. The size of the concrete slab on both sides was 460 mm × 300 mm × 150 mm, and the concrete strength grade was C40. Two layers of reinforcing mesh were set in the concrete slab, including four longitudinal steel bars and two stirrups. The grade of the longitudinal steel bar and stirrup was HRB400, with a diameter of 10. The steel beams were cast with C40 commercial concrete after rust removal, and the specimens were demolded after curing for 28 days under standard conditions (temperature: 20 ± 2 °C, relative humidity: no less than 95%). The studs had a ML-15 grade strength. The diameter and length of the stud were 16 and 80 mm, respectively. The specimens are shown in Figure 2. The surfaces of both the steel beam and reinforcement mesh were treated with anti-rust paint for rust prevention before the test.

### 2.2. Material Properties

After 28 days of curing, the average concrete cube strength and elastic modulus were 42.3 MPa and 3.31 × 10^4^ MPa, respectively. The material test results of the steel beam, stud, and reinforcement mesh are shown in Table 2.

### 2.3. Test Method

**Figure 3:**
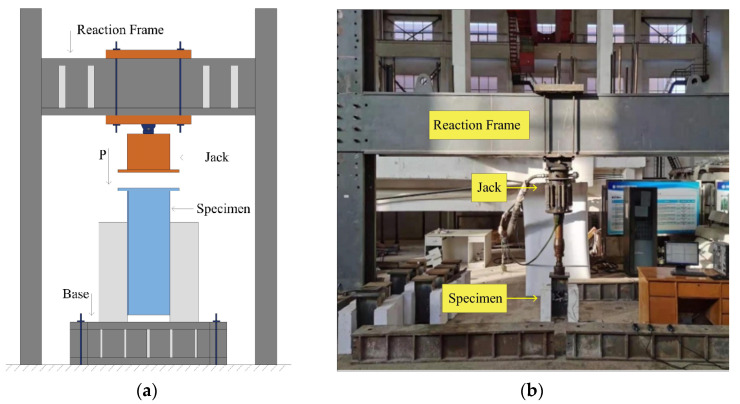
Test loading: (**a**) loading schematic; (**b**) site photo.

**Figure 4:**
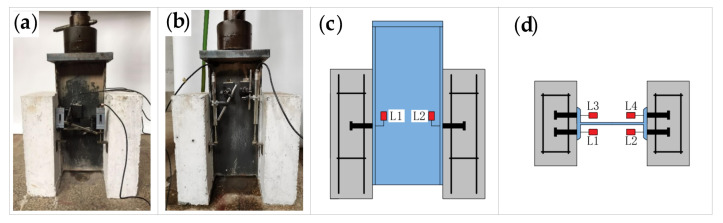
Arrangement of measurement points and displacement transducers: (**a**) static loading; (**b**) fatigue loading; (**c**) layout of measurement points: main view; (**d**) layout of measurement points: top view.

The test was carried out at the National Engineering Laboratory of High-speed Railway Construction Technology, Central South University. The loading device was a 500 kN hydraulic servo fatigue testing machine, as shown in Figure 3. Before loading, displacement transducers were installed on the horizontal surface, where the specimen studs were located, and two displacement transducers were placed on each side of specimen, as shown in Figure 4. The accuracy and range of the linear displacement transducer used for the static loading test were 0.001 mm and 30 mm, respectively, and the LVDT displacement transducer with the higher accuracy and sensitivity (L1, L2, L3, L4) was used for the fatigue test. Before the loading test, a layer of fine sand was laid under the two concrete slabs of the push-out specimen to ensure that the specimen was flat.

#### 2.3.1. Static Loading Test

Monotonic graded loading was adopted, starting with 10 kN as the load of each level, and the load for each level lasted for 10 min. When the load was increased to 50% of the ultimate load, each level of load was 5 kN, and when the load reached 80% of the ultimate load, the load level difference continued to decrease until the specimen was damaged. The measured data was automatically collected and recorded by the data acquisition instrument during the test.

#### 2.3.2. Fatigue Loading Test

For fatigue loading, the load varied sinusoidally, and the frequency of the fatigue testing machine was controlled at 4 Hz. The loading conditions of specimens F-1 to F-8 are shown in Table 3. Here, *p_u_* is the shear capacity of a single stud, which is obtained from the static loading test, and 25% and 50% of the shear capacity of a single stud are used as the minimum cyclic load *p*_min_ and the maximum cyclic load *p*_max_, respectively; this is based on theoretical calculation. Δ*P* is the range of the cyclic load. Specimens F-1 to F-7 were completely unloaded after a certain number of fatigue loading cycles, and then they were loaded to failure by static loading. However, the specimen F-8 was loaded to failure by fatigue loading.

## 3. Experimental Results and Discussion

### 3.1. Static Loading Test

#### 3.1.1. Failure Mode of the Specimens

**Figure 5:**
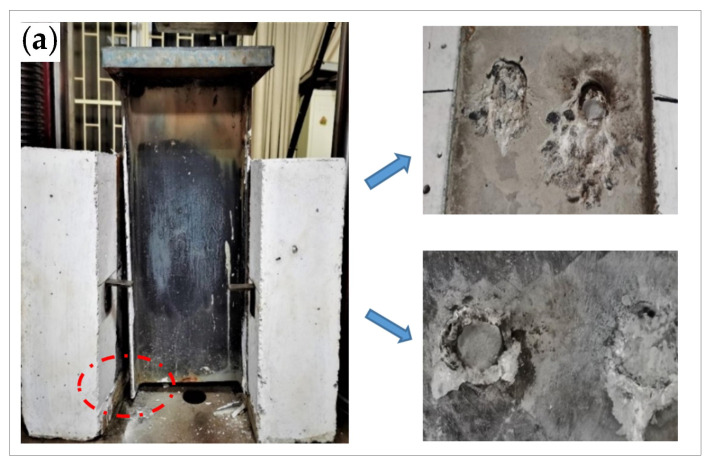

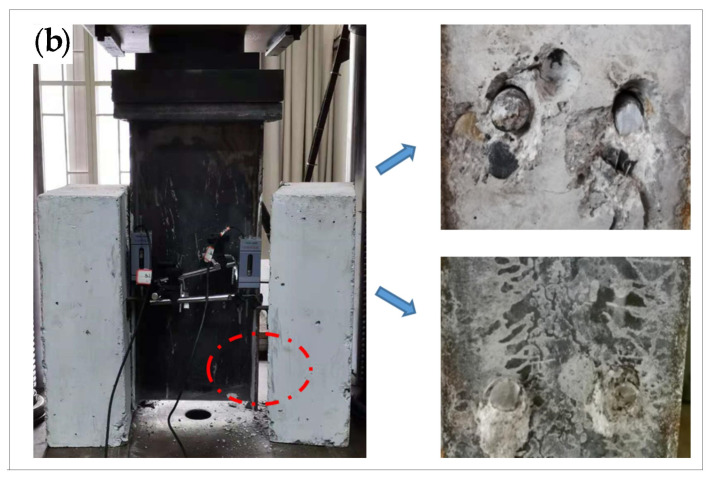
Static loading damage pattern of push-out specimen: (**a**) specimen S-1; (**b**) specimen S-2.

During the loading, the I-beam transferred most of the load to the studs, and the load of other parts was directly transferred to the concrete slab in the form of friction. The concrete under the root of the stud was deformed and cracked under the high stress, which caused relative deformation between the I-beam and the concrete. Both specimens S-1 and S-2 exhibit stud shear damage, as shown in Figure 5. The damage to the specimens was accompanied by a large ringing sound, while more cracks appeared on the surface of the concrete flange plate. The red circle in Figure 5 shows the damaged part of the specimen.

#### 3.1.2. Load-Slip Curve

**Figure 6:**
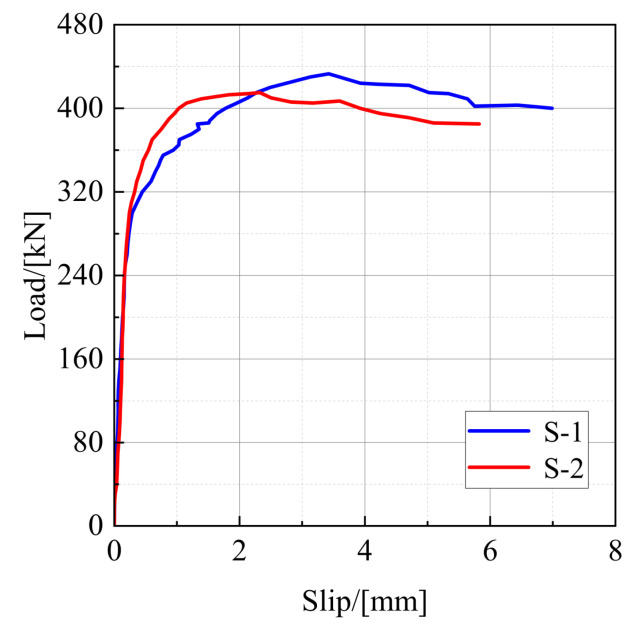
Load-slip curve of push-out specimens.

The results of the push-out static test are shown in Table 4. The average value measured by four displacement transducers is taken as the slip value of the push-out specimen, and the load-slip curves of specimens S-1 and S-2 are presented in Figure 6.

It can be seen from Figure 6 that the load-slip curve of the stud shear connector can be divided into three parts: elastic phase, elastic–plastic development phase, and plastic development phase. At the beginning of loading, the load is at a low level and the slip of the stud is small. Meanwhile, the load-slip curve of the push-out specimen is close to a straight line, and the stud is basically in the elastic stage. As the load increases, the stud enters the elastic–plastic working state, the stud shear stiffness decreases, and the slip value increases rapidly. When the load *P* reaches about 0.9 *P_u_*, the stud enters the plastic working state, while the load-slip curve becomes gentler, which means that the load enters a gentle increasing stage. However, the stud slip increases significantly, and the shear stiffness of the stud degrades continuously. Finally, the stud is sheared off, and the specimen is damaged.

### 3.2. Fatigue Loading Test

#### 3.2.1. Specimen Failure Mode

**Figure 7:**
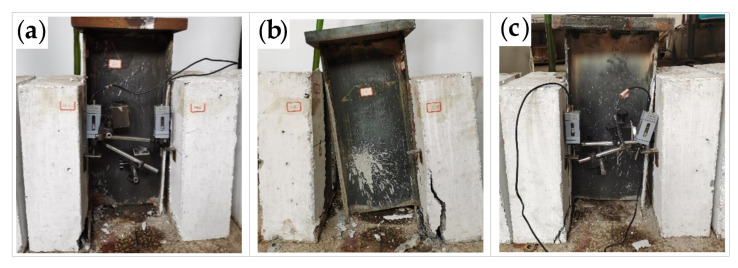
Shear fracture of studs in the concrete slab on one side of the specimen: (**a**) specimen F-1; (**b**) specimen F-4; (**c**) specimen F-5.

**Figure 8:**
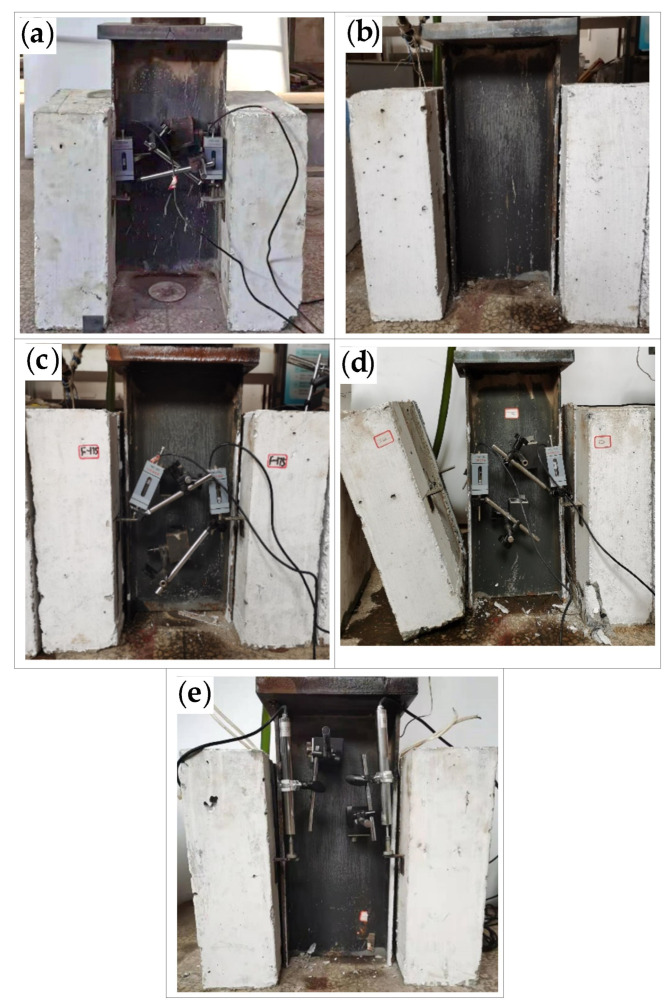
Shear fracture of studs in the concrete slab on both sides of the specimen: (**a**) specimen F-2; (**b**) specimen F-3; (**c**) specimen F-6; (**d**) specimen F-7; (**e**) specimen F-8.

The maximum and minimum cyclic loads were 216 and 108 kN, respectively. The specimens F-1 to F-7 were completely unloaded after a certain number of fatigue loading cycles, and then they were loaded to failure by static loading. On the other hand, the specimen F-8 was loaded to failure by fatigue loading. The failure modes of all the eight specimens are in accordance with the shear fracture of the studs. As shown in Figure 7, the studs are sheared off in the concrete slab on one side of specimens F-1, F-4, and F-5. On the other hand, as shown in Figure 8, the studs are sheared off in the concrete slab on both sides of specimens F-2, F-3, F-6, F-7, and F-8. Specimens F-1, F-4, and F-7 exhibit concrete spalling in the bottom area of the concrete slab during the loading process, while the other specimens do not show obvious cracks on the surface of the concrete slab during the fatigue loading process.

#### 3.2.2. Slip-Number of Fatigue Loading Cycle’s Curve

**Figure 9:**
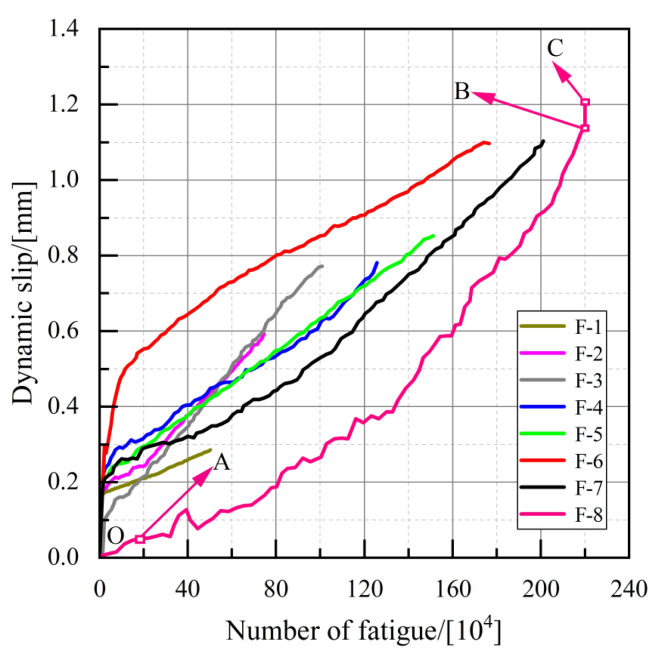
Slip-number of fatigue loading cycle’s curve.

The average of the measured values by the four displacement transducers is taken as the slip value of the fatigue specimen, and the slip-number of the fatigue loading cycle’s curve of each specimen is shown in Figure 9. The slip-number of the fatigue loading cycle’s curve of the push-out specimen can be divided into three stages: initial stage, middle stage, and final stage. Take specimen F-8 as an example; as can be seen in Figure 9, in the initial stage (OA), a small and fast-growing slip is produced. In the middle stage (AB), the fatigue crack of the stud expands with the increase in the number of loading cycles, and the relative slip between the steel plate and the concrete also begins to increase slowly. In the final stage (BC), due to the rapid expansion of the fatigue crack of the stud, the shear area of the stud decreases, and the stud cannot withstand the maximum cyclic load. Thus, the stud is instantaneously sheared off. The dynamic slip of the specimen grows slowly throughout the fatigue loading process, and the maximum slip is approximately 1.2 mm when fatigue damage occurs; this is much smaller than the ultimate slip value when static loading damage occurs.

#### 3.2.3. Fatigue Cracking Characteristics of Studs

**Figure 10:**
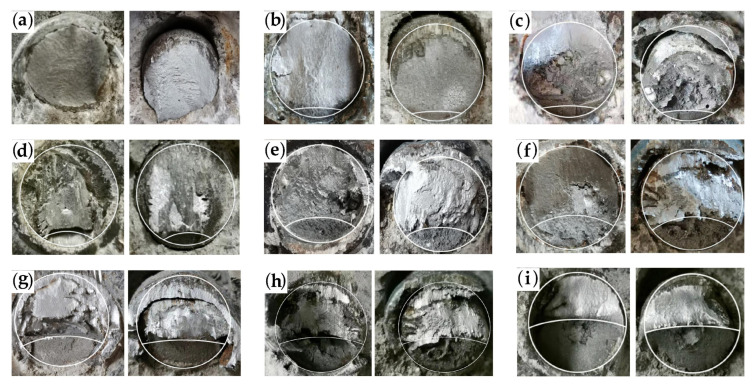
Cross-sectional characteristics of studs at different cyclic loads: (**a**) static damage; (**b**) specimen F-1; (**c**) specimen F-2; (**d**) specimen F-3; (**e**) specimen F-4; (**f**) specimen F-5; (**g**) specimen F-6; (**h**) specimen F-7; (**i**) specimen F-8.

Following the damage to the specimen, the stud section after shearing is shown in Figure 10. Here, for each specimen, the left figure depicts the fracture on the I-beam steel, while the right figure displays the stud fracture in the concrete. It is clear from Figure 10 that, when the damage is caused by static loading, the damaged section of the studs includes only one bright region. However, when the fatigue loading lasts for a certain number of cycles and then the specimen is loaded to failure by static loading, the damaged section of the stud can be obviously divided into two regions: the fatigue crack extension area with a dark color and the shear damage area with a bright color. Further, with the increase in the number of fatigue loading cycles, the radial crack extension depth of the stud increases, and the effective area of the stud gradually decreases; when complete fatigue failure occurs, the fatigue crack propagation area reaches about half of the stud section.

Following the damage to specimens F-1 to F-8, the crack depths of the studs, corresponding to different numbers of fatigue loading cycles, were measured. At the end of loading, the studs were removed from the specimen, and their cross-sectional photographs were taken. Then, the ratio of the area of the fatigue crack extension zone to the cross-sectional area of the studs was measured by CAD, in order to obtain the crack depth of the studs. The average value of the crack depth of the four studs was taken as the crack depth under the corresponding cyclic loads, and the results are shown in Table 5, where *a_c_* is the average crack depth value of the studs obtained from the test. The purpose of this work is to minimize the error, by taking the crack depth average of the four studs in the push-out specimen.

The theoretical fatigue crack depth *a_t_*, when fatigue damage occurs in the stud, can be calculated as follows [14]:

(1) $$a_{t}=d\left(1-\frac{P_{\max}}{Af_{u}}\right)$$

where *f_u_* is the ultimate tensile strength of the stud, *P*_max_ is the residual bearing capacity of the stud, shown in Table 6, *d* is the diameter of the stud, and *A* is the cross-sectional area of the stud.

**Figure 11:**
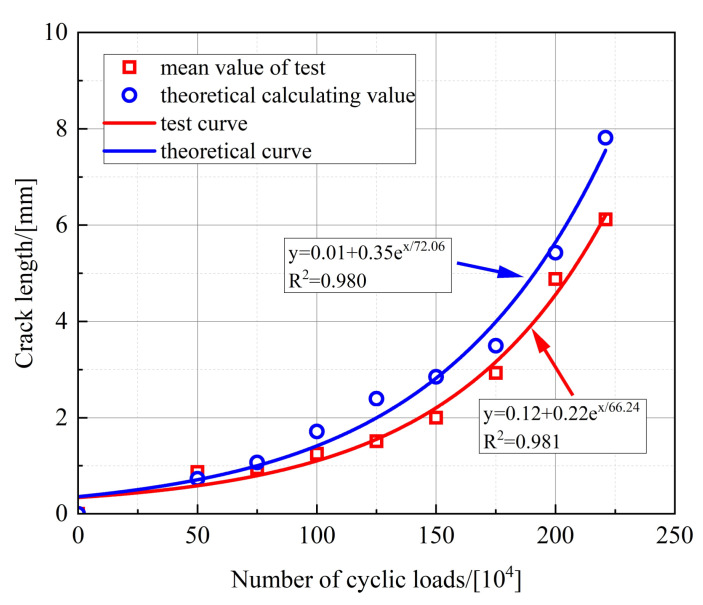
Variation in the crack depth with the number of fatigue loading cycles.

The calculated crack depth, when fatigue damage occurs in the stud, is shown in Table 5. It can be seen that the test and calculated values for the crack depth are very close to each other, and the relative error is small. Figure 11 shows the variation in the crack depth with the number of fatigue loading cycles. For the test and theoretical value, the initial crack depth of the stud is taken as 0 mm. The curve in Figure 11 is the result of the fitting, so the curve does not pass through the origin of the coordinates. It is clear that the test curves are in excellent agreement with the theoretical calculation curves. The fatigue crack depth increases exponentially with the increase in the number of fatigue loading cycles. The fatigue crack expansion of the studs is slow during the early stage of fatigue loading, and the fatigue cracks grow rapidly during the subsequent stage. Finally, the studs are damaged when they reach their fatigue life.

#### 3.2.4. Residual Bearing Capacity

**Figure 12:**
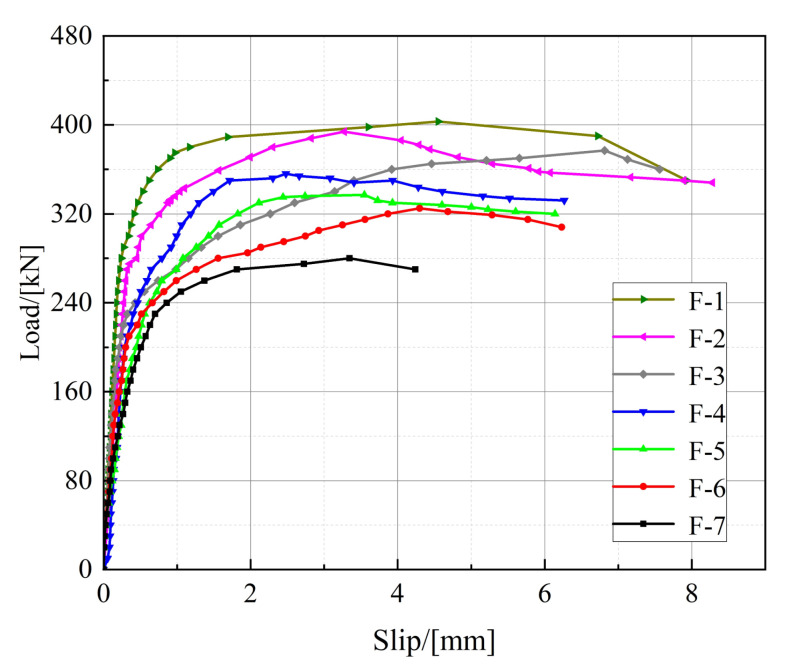
Load-slip curves of specimens under different cyclic loads.

The residual bearing capacity of the specimens is shown in Table 6. Here, F-8 is a complete fatigue loading specimen, whose residual bearing capacity is taken as the upper limit of fatigue loading. However, specimens F-1 to F-7 were completely unloaded after a certain number of fatigue loading cycles, and then they were loaded to failure by static loading; the residual bearing capacity of these specimens is taken as the peak load of static loading, and the load-slip curve of the reloaded specimen is shown in Figure 12. It is clear from Table 6 and Figure 12 that, during the static loading stage, the stiffness, ultimate slip, and ductility of the studs decrease with the increase in the fatigue load level; this is due to the fatigue accumulation damage.

### 3.3. Calculation Model for the Residual Bearing Capacity of Stud Shear Connectors

To circumvent the difficulty in measuring the fatigue extension area of studs in structure, the relationship between the ratio of the residual bearing capacity *P_u,N_* to the shear bearing capacity *P_u,_*_0_, and the ratio of fatigue crack depth to stud section diameter, is obtained through linear regression; this is to assess the residual bearing capacity of studs, in terms of the fatigue crack depth.

**Figure 13:**
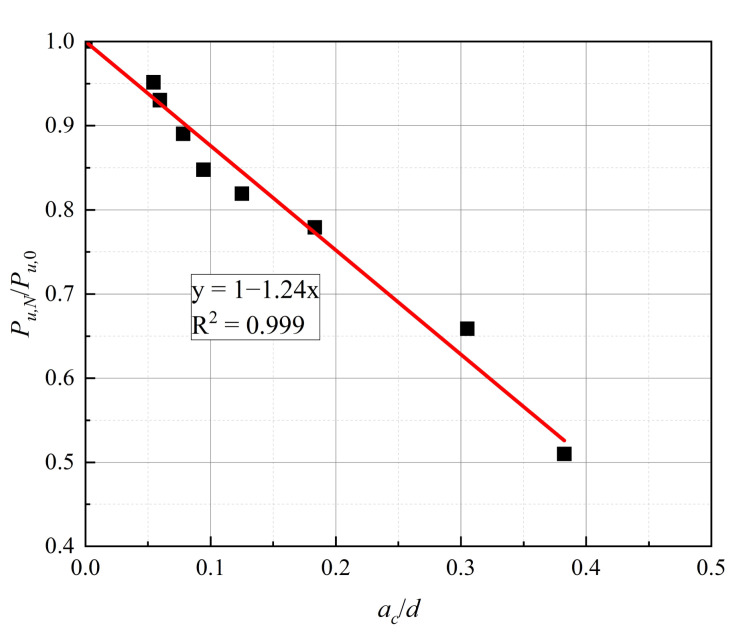
Relationship between *P_u,N_/P_u,_*_0_ and *a_c_/d*.

The relationship between *P_u,N_/P_u,_*_0_ and *a_c_/d* is shown in Figure 13. The slope of the fitting line is −1.24, and the goodness of fit is 0.999, which indicates a good fit. The residual bearing capacity of the studs is expressed as follows:

(2) $$\frac{P_{u,N}}{P_{u,0}}=1-1.24\frac{a_{c}}{d}$$

It can be seen from Equation (2) that the residual bearing capacity of the stud is negatively correlated with the fatigue crack depth. Specifically, as the fatigue crack depth increases, the residual bearing capacity of the stud decreases approximately linearly.

## 4. Simulation of Fatigue Crack Propagation Process of Stud Shear Connectors

**Figure 14:**
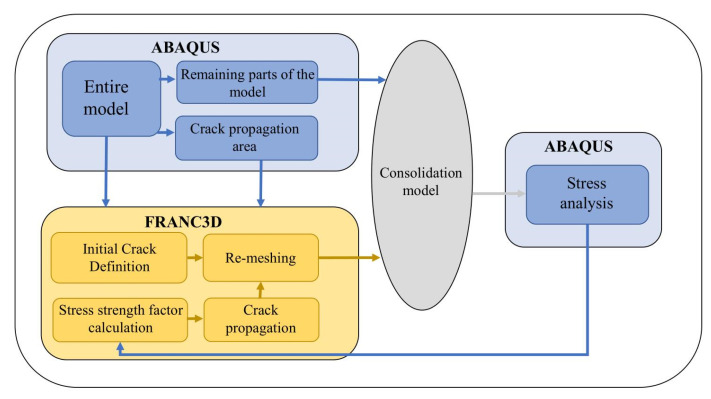
Fatigue crack propagation analysis.

The FEA software ABAQUS and three-dimensional (3D) crack growth simulation software FRANC3D were used to simulate the 3D fatigue crack propagation process of the stud. The calculation flow is shown in Figure 14.

### 4.1. ABAQUS Model Establishment

#### 4.1.1. Constitutive Relation Model of Materials

The plastic damage model is used for concrete, and the uniaxial tensile and compressive stress–strain relationships for concrete are selected from the constitutive relation, suggested in the Code for the Design of Concrete Structures (GB50010-2010) [15]. Further, the material parameters, used in the plastic damage model of concrete, are listed in Table 7, in which Ψ is the dilation angle, ϵ is the magnifying coefficient of eccentricity, f_b0_/f_c0_ is the yield stress ratio, K is the yield constant, and μ is the coefficient of viscosity. The ideal elastic–plastic model is used for both I-beam steel and rebar for the constitutive relation, and the trilinear model is used for the material of the studs [15]. The purpose of taking very small values for the viscosity coefficient during the simulation is to make the calculation more accurate.

#### 4.1.2. Finite Element Selection

The eight-node, 3D linear hexahedral solid element, with reduced integration (C3D8R), is used for concrete, I-beams, welding rings, and studs; meanwhile, the two-node, 3D truss unit (T3D2) is used for rebar. The dimensions of the welding ring are based on the “Cheese head studs for arc stud welding” (GB/T 10433-2002) [16], and the diameter and height of the welding ring are 21 and 4.5 mm, respectively. The same element type and constitutive relation model are used for the welding ring and the stud.

#### 4.1.3. Establishment of FEA Model

**Figure 15:**
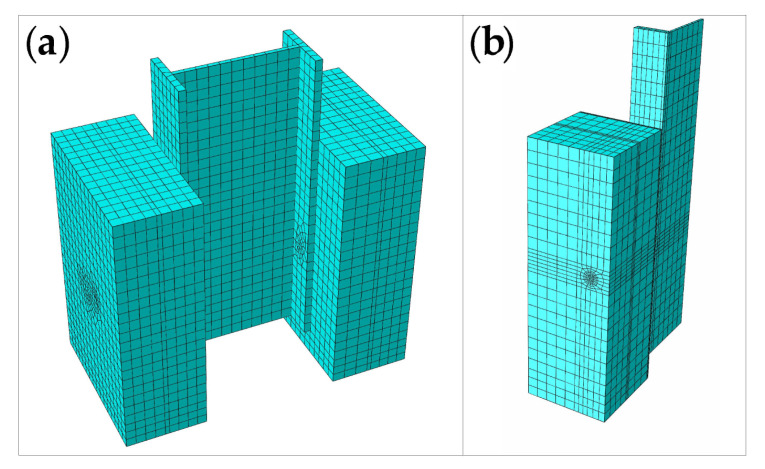
Finite element model of the push-out specimen: (**a**) entire model; (**b**) 1/4 model.

The welding ring element is merged with the I-beam element, and the overlapping surfaces of the welding ring element and the stud element are simulated by using a coupling node. The stud element and the I-beam element are connected by a tie. Surface-to-surface contact is used to simulate the friction between the studs and concrete, as well as between the I-beam steels and concrete, where the stiffness of studs and I-beam steels is larger; in addition, the contact surface is defined as the master surface, while the concrete contact surface is defined as the slave surface. Further, the tangential friction coefficient of the two pairs of contact surfaces is taken as 0.4, and hard contact is used in the normal direction [17]. A 1/4 model is used for simulation, and symmetric boundary conditions are applied on both of the symmetric planes. A fixed constraint is applied on the concrete bottom surface. The finite element model of the push-out specimen is shown in Figure 15. To obtain the descending section of the load-displacement curve, displacement loading is used for static loading. The mesh sizes of the I-beam, stud and concrete are about 20 mm, 2 mm and 20 mm on average, respectively. The time consumed during the simulation is several minutes.

### 4.2. Simulation Results and Discussion

**Figure 16:**
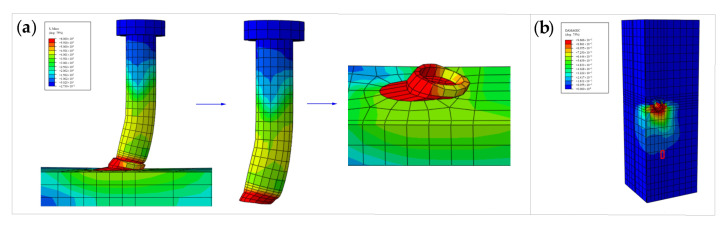
Stress cloud image of the specimen: (**a**) stud and welding ring; (**b**) concrete.

**Figure 17:**
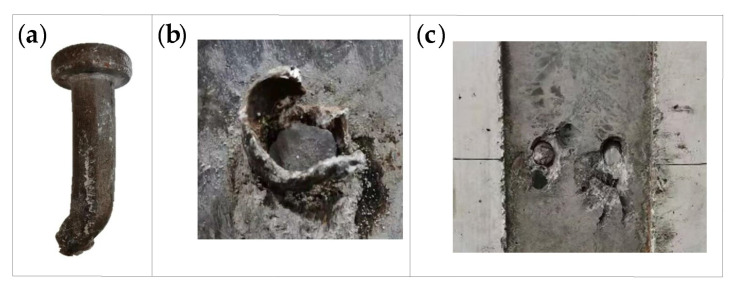
Static test failure mode of the push-out specimen: (**a**) stud; (**b**) welding ring; (**c**) concrete.

**Figure 18:**
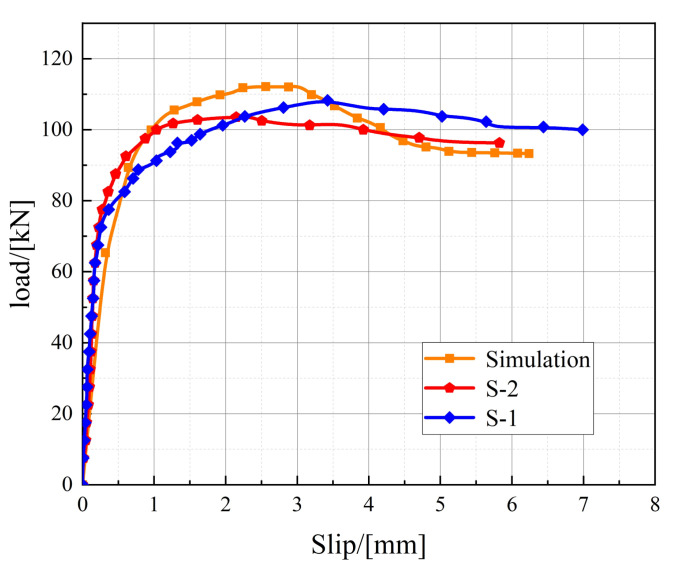
Comparison between the simulated and experimental load-slip curves.

The stress cloud images, at the time of static loading damage to the push-out specimens, are shown in Figure 16. The failure modes of the stud, welding ring, and concrete in the test, when the specimen is damaged, are shown in Figure 17. It can be seen from Figure 16 and Figure 17 that the stresses at the root of the stud and the welding ring are higher during the damage, and the stud root has an obvious radial deformation during the damage. Further, the deformation of the welding ring is large. At the same time, the concrete of the flange plate at the root of the stud in the middle of the specimen is deformed, cracked, and spalled under the high stress. The finite element simulation results are in good agreement with the test results. The bearing capacity and slip value of the studs, obtained by FEA, are shown in Table 8, and the load-slip curve of the studs are shown in Figure 18. There is friction between the steel beam and the concrete taking up part of the shear force at this stage, and there is some error between the interface friction in the finite element model and the actual one; therefore, there is a difference in the stiffness that causes the stiffness of the finite element model in the elastic section to be slightly lower than that of the test. It is evident from Figure 18 and Table 8 that the calculated and experimental load-displacement curves of the stud are in good agreement with each other. Through FEA, the bearing capacity, peak slip, and ultimate slip of the stud are obtained as 112.34 kN, 3.04 mm, and 6.04 mm, respectively, and the relative errors between the calculated (based on FEA) values and the test values of the above parameters are 6.10%, 5.92%, and 2.65%, respectively. This indicates that the simulated values for peak load, peak slip, and the ultimate slip of the stud are consistent with the experimental values.

### 4.3. Simulation Analysis of Fatigue Crack Propagation in Stud Shear Connectors

#### 4.3.1. Initial Crack Properties

**Figure 19:**
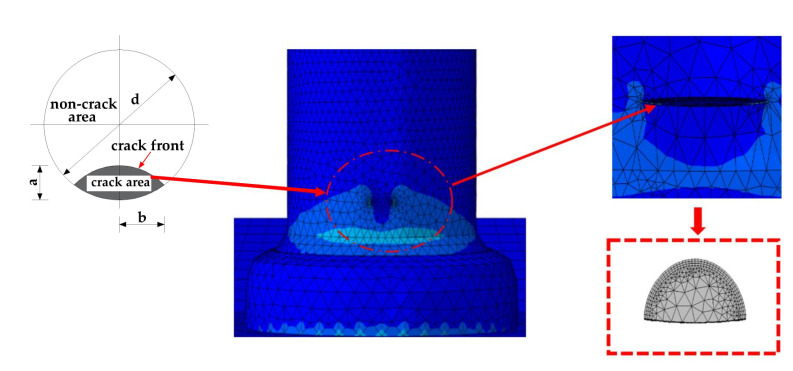
Initial crack characteristics.

Kala [18] obtained the following results based on the experimental results of Hudak and Tomica [19,20]: the initial crack depth *a*_0_ conformed to a normal distribution, with an arithmetic mean of 0.526 mm and a standard deviation of 0.504 mm. Therefore, for FEA, the initial crack depth of the stud is taken as 0.5 mm, and the initial crack is considered to have a semicircle shape with *a* = *b* = 0.5 mm. The initial crack shape and position are shown in Figure 19. After the initial crack is introduced, FRANC3D can automatically perform adaptive mesh re-division on the sub-model, and encrypt the mesh near the crack to improve the accuracy of calculating the stress intensity factor.

#### 4.3.2. Calculation of Stress Intensity Factor at the Leading Edge of the Crack

**Figure 20:**
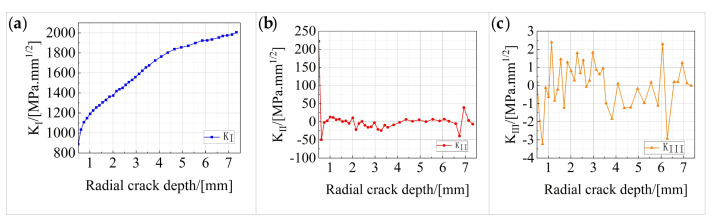
Variation in the stress intensity factors with the crack depth: (**a**) variation in K_I_ with the crack depth; (**b**) variation in K_II_ with the crack depth; (**c**) variation in K_III_ with the crack depth.

The stress intensity factor at the leading edge of the fatigue crack of the stud was calculated using M-integral, and the stress intensity factors for type I, type II, and type III crack patterns are K_I_, K_II_, and K_III_, respectively. The model was established after the initial crack was introduced at the root of the stud, and the crack stopped propagating when the stress intensity factor at the tip of the crack reached the fracture toughness value of the stud material. The median node of the stress intensity factor is selected, and the corresponding K_I_, K_II_, and K_III_ values were calculated for different crack sizes. The variation trends in the three stress intensity factors, with the crack depth, are shown in Figure 20.

It can be seen from Figure 20 that K_I_ is much larger than K_II_ and K_III_ during the entire fatigue crack propagation process, indicating that type I cracks play a dominant role in the fatigue crack propagation process of the studs. With the increase in the crack depth, the growth of K_I_ is fast in the beginning and slow later. Further, K_II_ is 221 MPa.mm^1/2^ in the early stage of crack propagation, and then it achieves a stable value of −50 to 50 MPa.mm^1/2^; this indicates that the initial fatigue crack has a larger torsion angle during the early propagation stage, and that the crack torsion angle is then gradually stabilized. K_III_ remains stable at −3 to 3 MPa.mm^1/2^ during the fatigue crack propagation process.

#### 4.3.3. Prediction of 3D Fatigue Crack Propagation

**Figure 21:**
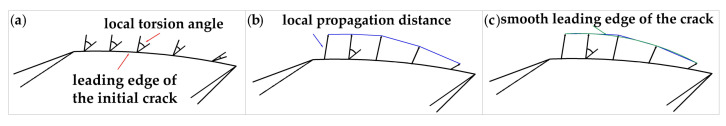
Fatigue crack propagation prediction: (**a**) local torsion angles determination; (**b**) local propagation distance determination; (**c**) smoothen the leading edge.

After calculating the stress intensity factor at the leading edge of the fatigue crack, the fatigue crack is updated in three steps [21], as shown in Figure 21. First, the local torsion angles of all the nodes on the leading edge of the fatigue crack are determined using the maximum stress criterion, and the local torsion angles of the nodes are fitted through polynomial regression to predict the fatigue crack propagation direction. Then, the local propagation distance of all the nodes on the leading edge of the crack is calculated by defining the crack propagation increment for each step. The crack propagation rate is low during the early stage of fatigue loading, and the crack propagation increment is taken as 10% of the crack characteristic length, in order to avoid the crack propagation interruption. When the crack length gradually increases, the crack propagation increment is taken as 15% of the crack characteristic length. Finally, during the crack propagation of the stud, the third-order polynomial is selected to smoothen the leading edge of the new crack after propagation, removing some points with large dispersion to make the leading-edge line of the new crack smoother. Subsequently, the fatigue crack always propagates according to the above three steps, and the modeling steps are repeated for fatigue crack propagation calculation; this is until the stress factor at the crack tip reaches the fracture toughness of the stud material. Then, the crack stops propagating, and the stud exhibits fatigue fracture.

After the fatigue crack is updated, the crack propagation rate model of Zhan [22] is selected to calculate the corresponding number of cyclic loads. Based on the fatigue test data, Zhan proposed a crack propagation rate model that considers the effect of stress ratio R on the fatigue life; the relevant equation is:

(3) $$\frac{da}{dN}=C{\left(e^{\alpha \cdot R}\cdot \Delta K\right)}^{m}$$

where *α* is a constant, whose value is taken as 0.65. *C* and *m* are material constants, whose values are determined by Huang’s model [23], and Δ*K* is the crack tip stress intensity factor amplitude.

**Figure 22:**
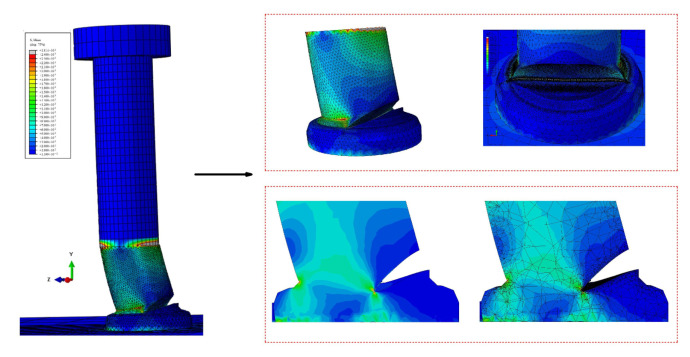
Fatigue crack pattern of studs.

The crack propagation pattern, corresponding to the fatigue damage to the stud, is shown in Figure 22.

**Figure 23:**
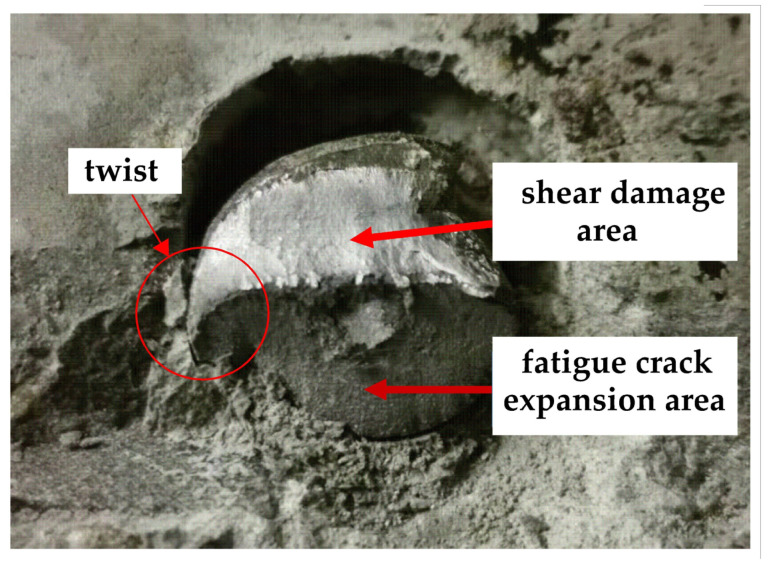
Cross-sectional characteristics of studs.

It can be seen from Figure 22 and Figure 23 that the crack is twisted during propagation. The reason for this twist in the simulation is that the studs in the test push-out specimen and the simulated push-out specimen model are accompanied by welding rings. The fatigue cracks in the studs in the test appear near the top of the welding rings; in addition, and the fatigue cracks in the studs do not always propagate in the same plane, but gradually deflect during propagation until the studs reach shear failure. The fatigue life of the stud, obtained by FRANC3D software simulation, is 2,307,041 cycles, and the test fatigue life of the stud is 2.21 million cycles; therefore, the relative error between the FEA value and the test value is 4.39%. Additionally, the fatigue crack depth is nearly half of the stud diameter, which is 7.33 mm, when the fatigue damage occurs. The test and theoretically-calculated values for the crack depth, when fatigue damage occurs in the studs, are 6.12 mm and 7.81 mm, respectively. The FEA model is established [24] and the results are in good agreement with the test and theoretically calculated values, which indicates that the fatigue crack propagation FEA model of the stud is valid and reasonable.

**Figure 24:**
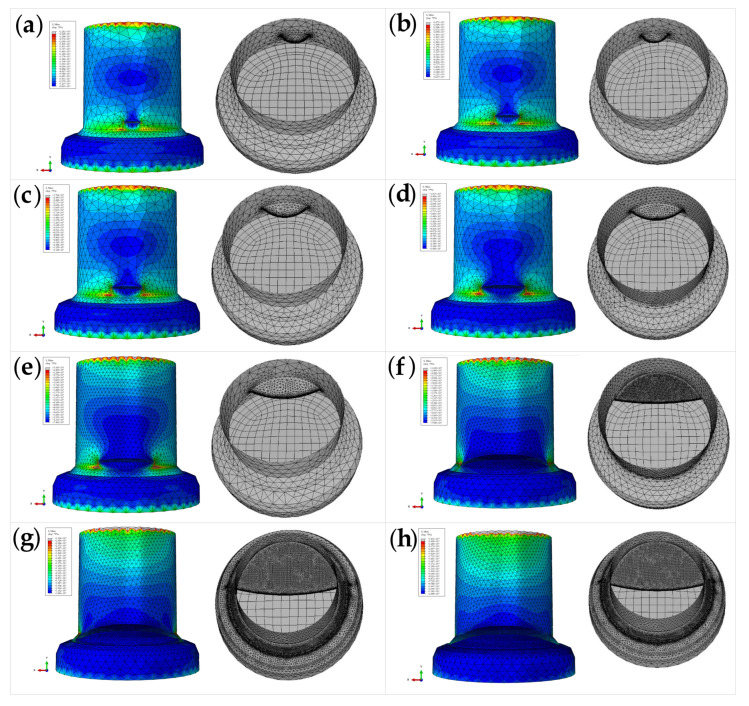
Crack patterns of studs under different numbers of cyclic loads: (**a**) N = 500,000; (**b**) N = 750,000; (**c**) N = 1,000,000; (**d**) N = 1,250,000; (**e**) N = 1,500,000; (**f**) N = 1,750,000; (**g**) N = 2,000,000; (**h**) N = 2,210,000.

**Figure 25:**
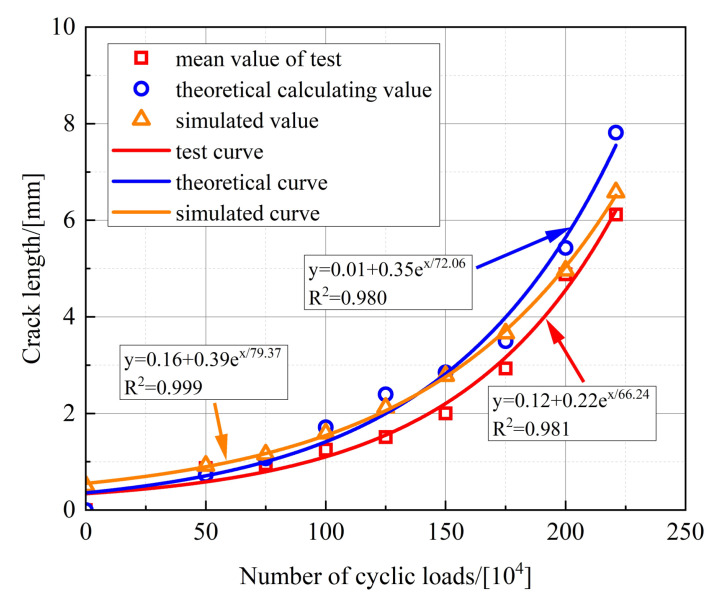
Comparison of simulated, theoretical, and test curves.

Based on the stress intensity factor data of the nodes during each step of fatigue crack propagation, the corresponding number of cyclic loads is calculated, and the crack propagation pattern of the studs at different cyclic loads is shown in Figure 24. It can be seen that the crack depth gradually increases in the circumferential direction with the increase in the number of cyclic loads. The corresponding radial crack depths, under different cyclic loads, are presented in Table 9, and the relationship between the number of cyclic loads and the fatigue crack depth is shown in Figure 25.

For FEA, the initial crack depth of the stud is taken as 0.5 mm, so the simulated curve has different intercepts from the test curve and the theoretical curve. It can be seen from Figure 25 that the relationship curves between the fatigue crack depth of studs and the number of cyclic loads, obtained through the FEA, experimental test, and theoretical calculation, are basically consistent. Therefore, the entire fatigue crack propagation process of the studs can be effectively simulated by using the ABAQUS and FRANC3D software concurrently.

## 5. Conclusions

In this work, the specimen was completely unloaded after a certain number of fatigue loading cycles, and then loaded statically until the specimen was damaged. The fatigue properties, i.e., the failure mode, the dynamic slip-fatigue number curve, the cross-sectional characteristics, and the residual bearing capacity of the stud specimens, were systematically studied. Then, combined with the ABAQUS and FRANC3D software, the fatigue crack propagation FEA model of the stud was established. Finally, the three-dimensional fatigue crack propagation model was established. The conclusions of this work are as follows.:

- Irrespective of static loading or fatigue loading, the damage to the push-out specimen was caused by the shear damage to the stud root. The section of the stud could be divided into a dark fatigue crack expansion area and a bright shear damage area; with the increase in the number of loading cycles, the fatigue crack expansion area also gradually expanded. The dynamic slip during fatigue loading initially grew faster because the stud compacted the surrounding concrete, but when the number of fatigue loading cycles exceeded 100,000, it increased approximately linearly with the increase in the number of fatigue loading cycles.
- Based on the crack size formula in fracture mechanics, an effective model was established for calculating the residual bearing capacity of the stud.
- As the number of fatigue loading cycles increased, the stiffness, ultimate slip, and ductility of the studs decreased. Further, the relative value of the fatigue crack extension area increased, while the relative value of the residual bearing capacity of the studs decreased approximately linearly; this indicates that the linear damage theory could be used to analyze the fatigue damage to the stud shear connectors.
- An FEA model for fatigue crack propagation in the stud was established by combining ABAQUS and FRANC3D software. The FEA results showed that the crack twisted during the propagation process, and the fatigue crack depth of the stud was 7.33 mm when the fatigue damage occurred. The test and theoretically calculated values of the crack depth of the stud during the fatigue damage were 6.12 and 7.81 mm, respectively. The simulation values based on FEA were in good agreement with the test and theoretically calculated values.
- The relationship curves between the fatigue crack depth curves of the studs and the number of cyclic loads, obtained through the finite element simulation, experimental test, and theoretical calculation, were in good agreement with each other. The fatigue crack depth increased exponentially with the increase in the number of cyclic loads. The fatigue crack propagation rate of the studs was low at the beginning of fatigue loading, and the fatigue cracks grew rapidly during the later stage. Finally, the studs fractured when they reached their fatigue life.

In conclusion, the three-dimensional fatigue crack propagation model, established in this paper, can accurately simulate the whole process of the fatigue crack propagation of studs. However, in the actual engineering structure, the stud shear connectors are mainly subjected to variable amplitude fatigue loading, and most of the tests are currently using equal amplitude fatigue loading. There is less research on the effect of initial defects on the fatigue life of studs. Therefore, the effects of variable amplitude loading and the initial defects of studs, should be considered in the subsequent study of the fatigue crack propagation of stud shear connectors.

## Figures and Tables

**Table 1:** Abbreviation table.

Full Name	Abbreviation
finite element analysis	FEA
shear capacity of a single stud	*p_u_*
minimum cyclic load	*p_min_*
maximum cyclic load	*p_max_*
range of cyclic load	Δ*P*
peak load	*P_u_*
peak slip	*S_u_*
ultimate slip	*S_max_*
load	*P*
the average value of the crack depths of the studs obtained from the test	*a_c_*
the theoretical fatigue crack depth	*a_t_*
the ultimate tensile strength of the stud	*f_u_*
the residual bearing capacity of the studs	*P_max_*
the diameter of the stud	*d*
the cross-sectional area of the stud	*A*
the value of the crack depths of a stud in specimen obtained from the test	*a_1_, a_2_, a_3_, a_4_*
the residual load capacity of a single stud	*P_u,N_*
the shear bearing capacity of a single stud	*P_u,0_*
the goodness of fit	*R^2^*
dilation angle	Ψ
magnifying coefficient of eccentricity	ϵ
yield stress ratio	*f_b0_/f_c0_*
yield constant	K
coefficient of viscosity	μ
the stress intensity factors for type I	K_I_
the stress intensity factors for type II	K_II_
the stress intensity factors for type III	K_III_
material constants	C
material constants	m
the crack tip stress intensity factor amplitude	Δ*K*
stress ratio	*R*
the numbers of cyclic loads	N

**Table 2:** Material test results.

Project	Yield Strength (MPa)	Ultimate Strength (MPa)	Modulus of Elasticity (MPa)
I-beam steel	362	458	2.10 × 10^5^
Steel bar	400	570	2.06 × 10^5^
Stud shear connector	442	525	2.06 × 10^5^

**Table 3:** Fatigue loading test conditions.

Specimen	*p*_max_/*p_u_*	*p*_min_/*p_u_*	Δ*P*/*p_u_*	Number of Fatigue (10^4^)
F-1	0.5	0.25	0.25	50
F-2				75
F-3				100
F-4				125
F-5				150
F-6				175
F-7				200
F-8				Complete fatigue (221)

**Table 4:** Static loading test results of specimens.

Specimen	Load Capacity of a Single Stud	Peak Load *P_u_* (kN)	Peak Slip *S_u_* (mm)	Ultimate Slip *S*_max_ (mm)	Failure Mode
S-1	108.25	433	3.42	6.98	One side of the stud sheared off
S-2	103.50	414	2.32	5.83	

**Table 5:** Crack depth under different cyclic loads.

Specimen	Number of Fatigue (10^4^)	*a_1_* (mm)	*a_2_* (mm)	*a_3_* (mm)	*a_4_* (mm)	*a_c_* (mm)	*a_t_* (mm)
F-1	50	0.50	0	1.45	1.53	0.87	0.73
F-2	75	1.75	1.20	0.47	0.38	0.95	1.07
F-3	100	1.95	1.65	0.75	0.65	1.25	1.71
F-4	125	3.78	1.96	0	0.30	1.51	2.40
F-5	150	0.62	0.40	4.37	2.61	2.00	2.85
F-6	175	5.94	3.11	1.36	1.31	2.93	3.49
F-7	200	4.66	3.86	5.19	5.81	4.88	5.43
F-8	221	5.75	6.64	6.13	5.96	6.12	7.81

**Table 6:** Residual bearing capacity and fatigue crack depth of specimens under different cyclic loads.

Specimen	Number of Fatigue (10^4^)	Residual Load Capacity (kN)	Residual Load Capacity of a Single Stud *P_u,N_* (kN)	*P_u,N_*/*P_u,_*_0_	*a_c_* (mm)	*a_c_/d*
Mean of S-1 and S-2	0	423.50	105.88	1.00	0	0
F-1	50	403.00	100.75	0.95	0.87	0.05
F-2	75	394.00	98.50	0.93	0.95	0.06
F-3	100	377.00	94.25	0.89	1.25	0.08
F-4	125	359.00	89.75	0.85	1.51	0.09
F-5	150	347.00	86.75	0.82	2.00	0.13
F-6	175	330.00	82.50	0.78	2.93	0.18
F-7	200	279.00	69.75	0.66	4.88	0.31
F-8	221	216.00	54.00	0.51	6.12	0.38

**Table 7:** Concrete material parameters.

Ψ	ϵ	f_b0_/f_c0_	K	μ
36°	0.1	1.16	0.6667	0.00001

**Table 8:** Comparison between the simulated and experimental result.

Project	Test Value		Average Value of Test	Simulated Value	Relative Error/%
Peak load *P_u_* (kN)	108.25	103.50	105.88	112.34	6.10
Peak slip *S_u_* (mm)	3.42	2.32	2.87	3.04	5.92
Ultimate slip *S*_max_ (mm)	6.98	5.83	6.41	6.24	2.65

**Table 9:** Comparison of FEA results with the test and theoretical calculation results.

Number of Cyclic Loads (10^4^)	Crack Depth of Test (mm)	Crack Depth of Theoretics (mm)	Crack Depth of Simulation (mm)
50	0.87	0.73	0.92
75	0.95	1.07	1.15
100	1.25	1.71	1.59
125	1.53	2.40	2.12
150	2.15	2.85	2.78
175	2.93	3.49	3.66
200	4.88	5.43	4.96
221	6.12	7.81	6.58

## Data Availability

The data provided in this study could be released upon reasonable request.

## References

[1] Zhu T., Wang J., Yiu H. (2007). Characteristic summarization of headed stud shear connectors in steel and concrete composite beams. Jiangsu Constr..

[2] Chen L., Jiang S., Zhao J. (2010). The review on bearing capacity of stud shear connectors. Steel Constr..

[3] Xu J., Sun H., Chen W. (2021). Experiment-based fatigue behaviors and damage detection study of headed shear studs in steel–concrete composite beams. Appl. Sci..

[4] Huang Q., Wang B., Liu X. (2018). Residual mechanical properties of stud connectors under cyclic loading. J. South China Univ. Technol..

[5] Hanswille G., Porsch M., Ustundag C. (2007). Resistance of headed studs subjected to fatigue loading: Part I: Experimental study. J. Constr. Steel Res..

[6] Liu T., Nie X., Zeng J. (2022). Static and fatigue behaviors of corroded stud connectors in weathering steel–concrete composite beams. Eng. Struct..

[7] Wei C., Zhang Q., Zhou Y. (2022). Static and fatigue behaviors of short stud connectors embedded in ultra-high performance concrete. Eng. Struct..

[8] Wang D., Tan B., Xiang S. (2022). Fatigue Crack Propagation and Life Analysis of Stud Connectors in Steel-Concrete Composite Structures. Sustainability.

[9] Liang X., Yi X., Wang B. (2022). Slip behavior of stud connectors of steel-concrete composite beams in the whole process of fatigue loading. Structures.

[10] Walker K. (1970). Effects of Environment and Complex Load History on Fatigue Life.

[11] Kujawski D. (2001). A fatigue crack driving force parameter with load ratio effects. Int. J. Fatigue.

[12] Ewalds H.L., Wanhill R. (1985). Fracture Mechanics.

[13] Johnson R.P., Anderson D. (2004). Eurocode4: Design of Composite Steel and Concrete Structures. Part 1.1: General Rules and Rules for Buildings.

[14] Wang Y., Nie J. (2009). Fatigue behavior of studs in a composite beam based on fracture mechanics. J. Tsinghua Univ. (Sci. Technol.).

[15] (2011). Code for Design of Concrete Structures.

[16] (2002). Cheese Head Studs for Arc Stud Welding.

[17] Liu Y. (2019). Fatigue Failure Mechanism of Steel-High Performance Concrete Composite Bridge Deck with Large-Size U-Ribs. Ph.D. Dissertation.

[18] Kala Z. (2006). Sensitivity analysis of fatigue behaviour of steel structure under in-plane bending. Nonlinear Anal. Model. Control..

[19] Hudak S.J., McClung R.C., Bartlett M.L. (1990). A Comparison of Single-Cycle Versus Multiple-Cycle Proof Testing Strategies.

[20] Tomica V. Key Dimensions of Fatigue Cracks in Steel Structures. Proceedings of the 20th Czech-Slovak Conference with International Participation, Steel Structures and Bridges.

[21] Li F.Z., Shih C.F., Needleman A. (1985). A comparison of methods for calculating energy release rates. Eng. Fract. Mech..

[22] Zhan W., Lu N., Zhang C. (2014). A new approximate model for the R-ratio effect on fatigue crack growth rate. Eng. Fract. Mech..

[23] Huang X., Moan T. (2007). Improved modeling of the effect of R-ratio on crack growth rate. Int. J. Fatigue.

[24] Ding F.X., Wu X., Xiang P., Yu Z.W. (2021). New Damage Ratio Strength Criterion for Concrete and Lightweight Aggregate Concrete. ACI Struct. J..

